# Automated identification of unknown decedents: matching postmortem CT images with clinical databases

**DOI:** 10.1007/s00414-025-03528-9

**Published:** 2025-05-24

**Authors:** Andreas Heinrich, Michael Hubig, Ulf Teichgräber, Gita Mall

**Affiliations:** 1 Department of Radiology, Jena University Hospital– Friedrich Schiller University, Am Klinikum 1, 07747 Jena, Germany; 2 Institute of Forensic Medicine, Jena University Hospital– Friedrich Schiller University, Am Klinikum 1, 07747 Jena, Germany

**Keywords:** Computer vision systems, Computed tomography, X-ray, Maxillary sinus, Human identification

## Abstract

The maxillary sinus plays an important role in the forensic identification of unknown deceased individuals. This study aimed to evaluate whether matching postmortem computed tomography (CT) images from virtual autopsies with antemortem CT examinations from a clinical database enables reliable identification using computer vision (CV) techniques. From ten virtual autopsies, CT images of the maxillary sinus were selected for comparison against 853 antemortem CT examinations from 738 individuals. A total of 60,255 antemortem CT slices underwent image processing, CV feature extraction, and were stored in an antemortem CV database. The number of matching points between CV features of the postmortem image and the antemortem reference image served as an indicator of identification accuracy. The identification rate was 50% (5/10) at rank 1 (with the sought identity having the highest number of matching points), 80% (8/10) at rank 2, and 100% (10/10) at rank 7 among the 738 potential identities. Challenges were observed when the antemortem reference CT examination depicted only parts of the maxillary sinus or when injuries were present. Additionally, postmortem imaging should closely replicate antemortem imaging standards to maximize the number of CV matching points. In conclusion, the findings suggest that it is feasible to identify individuals using postmortem CT images from virtual autopsies in combination with a clinical database. However, postmortem imaging should ideally adhere to clinical imaging standards to achieve more CV matching points for the sought identity with the antemortem reference.

## Introduction

Natural disasters, terrorist attacks, war, severe accidents, migration, or homelessness can result in the discovery of unknown deceased individuals [1, 2]. In these cases, identification can be a significant challenge. Computed tomography (CT) serves as a non-invasive tool for detailed anatomical examination and evidence collection in forensic investigations [3–5]. Additionally, the use of CT data for the automated identification of unknown deceased individuals could be a novel and particularly valuable approach when dealing with a large number of individuals that need to be identified. Previous studies [2, 6–9] demonstrated the utility of paranasal sinuses for accurate personal identification from CT slices.

A new personal identification method [10] based on computer vision (CV) has demonstrated success in identifying individuals using single CT slices, particularly by analyzing the maxillary sinuses. This approach extracts distinctive features from cranial CT images - patterns that are unique to each individual. These CV features are compared between the CT image of an unknown individual and a database of antemortem CV features. When similar CV features are identified in both images, they are matched to form matching points. The number of matching points indicates the likelihood that the unknown individual corresponds to a specific identity in the database. While effective, this method has so far been applied only to comparisons of antemortem data and relies on individual CT slices rather than entire CT series. Its applicability to postmortem data remains unexplored.

This study aims to evaluate whether the automatic CV-based personal identification method is suitable for postmortem data by matching postmortem CT images with complete antemortem CT series from a clinical database. This approach would simplify the process, as the antemortem data would not require prior analysis or filtering.

## Materials and methods

The study was approved by the local institutional review board (IRB) at Jena University Hospital (registration number 2019-1505-MV) and conducted in accordance with the Declaration of Helsinki and applicable ethical standards for retrospective research using pseudonymized postmortem and antemortem data. Written informed consent was not required for either living patients or deceased individuals, as the study was based on pseudonymized CT data. No identifying characteristics of the individuals studied were included in the publication. Human Ethics and Consent to Participate declarations: not applicable.

This retrospective study analyzed available virtual autopsies that had at least one antemortem reference CT examination of the head. Another inclusion criterion was the visualization of the maxillary sinuses on both antemortem and postmortem CT images, allowing a total of 10 virtual autopsies to be included in this study. The selection process was based on postmortem examinations identified under the respective procedure codes in the picture archiving and communication system (PACS) between 2012 and 2024. After identifying potential cases, the availability of antemortem reference scans was assessed, followed by a final evaluation of the inclusion criteria. The data were pseudonymized after export from the PACS to ensure no direct linkage to individual persons. The 10 individuals (ages 38–89 years, mean age 65.89 ± 19.13 years, 8 male) had a combined total of 22 antemortem head CT series. Of these, six individuals had only one reference CT series each, while one individual had two series, another had three, one had four, and one had seven series. The CT protocol parameters for all CT series are shown in Table 1.

**Table 1:** CT parameters with their frequencies (in parentheses) for the postmortem and antemortem series of the sought identity, as well as for the antemortem CV database (corresponding to other individuals and the 22 antemortem series of the sought identities)

	Sought identity		CV database	
	Postmortem	Antemortem	Antemortem	
Voltage [kVp]	120 (9x) 140 (1x)	120 (21x) 140 (1x)	120 (851x) 140 (2x)	
Current [mA]	344.50 ± 108.28	263.24 ± 97.30	90.86 ± 37.84	
Exposure [mAs]	7.70 ± 4.69	9.00 ± 11.75	10.19 ± 2.97	
Scan mode	helical (9x) axial (1x)	helical (7x) axial (15x)	helical (838x) axial (15x)	
Slice thickness [mm]	2.5 (1x) 0.625 (9x)	5 (10x) 2.5 (10x) 3 (1x) 0.625 (1x)	5 (10x) 2.5 (841x) 3 (1x) 0.625 (1x)	

### Signal processing, CV feature extraction and matching process

**Fig. 1:**
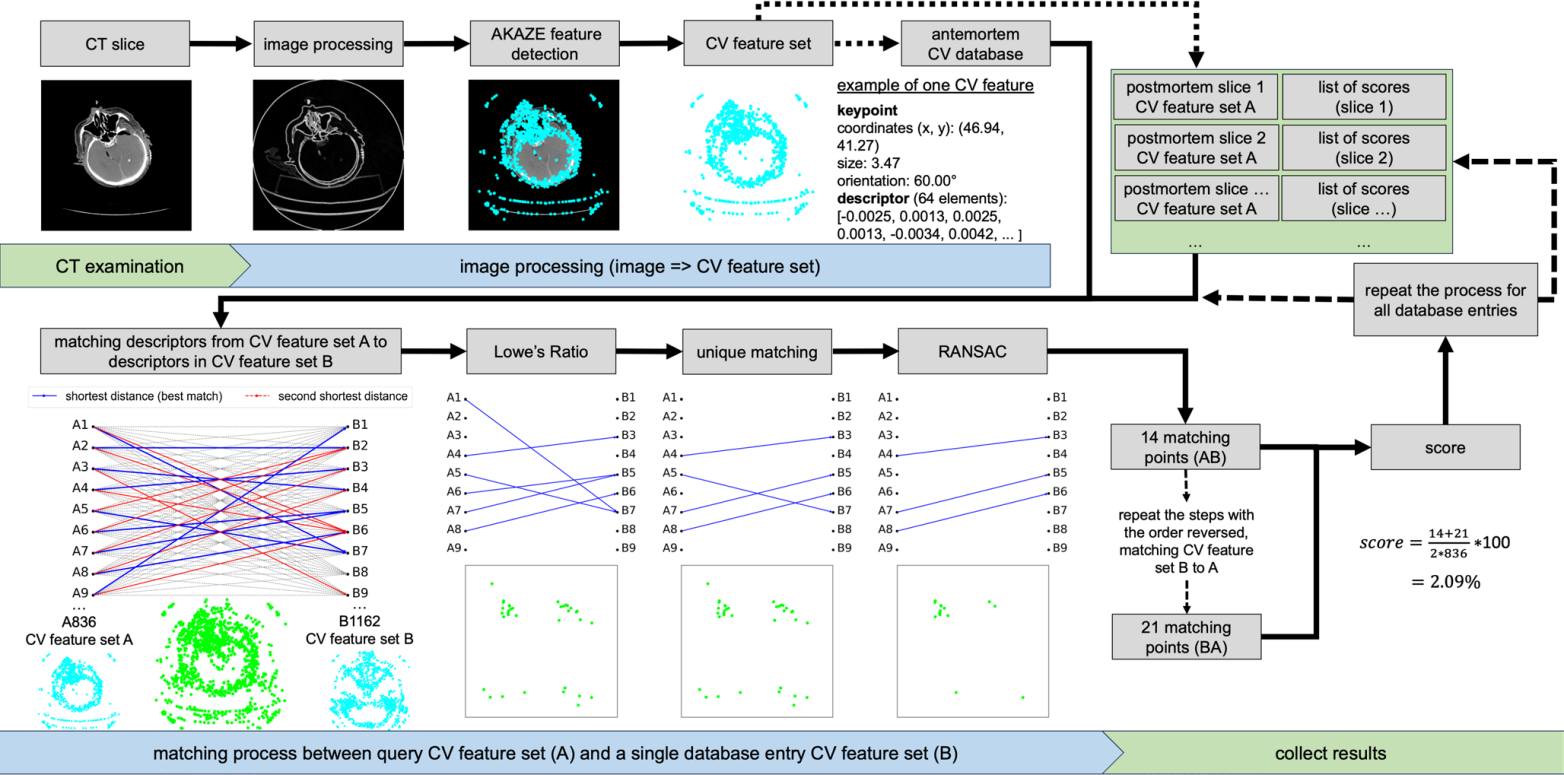
The figure illustrates the steps involved in CV-based personal identification. First, the CT slices undergo image preprocessing, including edge enhancement, an averaging filter, and 8-bit normalization. These processed images are then used for feature detection with the AKAZE algorithm, which identifies keypoints and their corresponding descriptors, forming the CV feature set. This CV feature set can be used independently of the original image, for example, by storing it in the antemortem database or using it as a postmortem query set for comparison with the database. In the matching process, each descriptor from the postmortem query CV feature set (**A**) is compared to all descriptors in the reference CV feature set (**B**) of a single database entry based on the measurement of squared Euclidean distance. For each descriptor in A, the closest match (shortest distance) and the second-closest match in B are determined. Each descriptor in A is linked to its closest match in B, while not all descriptors in B need to be assigned to A. A single descriptor in B can be associated with multiple descriptors in A. Lowe’s ratio test is then applied by comparing the distance between the closest and the second-closest match. Only those matches with a sufficiently low ratio are kept, ensuring that the most reliable matches are retained. Furthermore, each descriptor in B can be assigned to at most one descriptor in A to ensure a unique matching, where only the best result (closest match) is kept. Finally, RANSAC is used to remove outliers by iteratively selecting random subsets of matches and refining the transformation, ensuring that only the most consistent matches remain, which enhances the overall robustness of the matching process. At the end, a number of matching points is obtained. This process is then repeated with the order of the CV feature sets reversed. A score is calculated from both runs by dividing the average number of matching points by the total CV features in set A, and it is stored in a list of scores for the query set

The method follows the approach used in [10] and is briefly summarized here. Figure 1 shows a flowchart of the method, starting with the extraction of the CV feature sets (one set per image), where the antemortem sets are stored in the CV database and the postmortem set is used for comparison with the antemortem sets in the CV database. Each CT image underwent image processing, including color depth normalization to 8 bits, edge enhancement using modified Sobel filters, and noise reduction with an averaging filter. Next, the accelerated KAZE (AKAZE) algorithm [11, 12] was applied to automatically detect and extract CV features from the image. It works by finding keypoints, which are distinctive points where there is a noticeable change in intensity, such as edges or corners. To detect these keypoints, AKAZE uses the Hessian matrix, which analyzes the image’s second derivatives to identify points where the intensity changes most significantly. The algorithm looks for these points at multiple levels of image detail, or scales, ensuring that the keypoints are stable and significant. Once keypoints are found, AKAZE creates a descriptor for each one, which is a mathematical summary of the area around it - essentially a “fingerprint” of the feature that captures its unique local structure. This descriptor allows the keypoint to be identified in other images, even if they are rotated, resized, or otherwise transformed. For the antemortem data, the detected CV feature sets, along with metadata (study date, birth date, sex, and patient ID) from the Digital Imaging and Communications in Medicine (DICOM) header, were stored in an encrypted antemortem CV database.

During the matching process, a postmortem CV feature set (representing one image of an unknown individual) is used as the query set and compared to the CV features in the antemortem CV database. For each descriptor in the query set, the closest matches are identified by calculating the squared Euclidean distance to the descriptors in the reference set (database entry). Lowe’s ratio test is then applied to ensure that only reliable matches are retained, by verifying that the distance to the closest match is much smaller than the distance to the second closest one. Additionally, each descriptor can have at most one match, ensuring unique pairings of matching points. To improve accuracy, the Random Sample Consensus (RANSAC) algorithm is used to eliminate incorrect matches. RANSAC works by testing various scenarios to identify the best-fitting model and removing outliers.

The final similarity score, which ranges from 0% (no match) to 100% (identical image), is calculated by normalizing the number of matching points relative to the total number of CV features in the query set. The matching process is performed in both directions: first by comparing the query set (A) to a database entry (B), and then in reverse (B to A). The final score is the average of the results from both directions:

1 $$\eqalign{& score = {{{\rm{matching}}\_{\rm{point}}{{\rm{s}}_{{\rm{AB}}}}{\rm{ + matching}}\_{\rm{point}}{{\rm{s}}_{{\rm{BA}}}}} \over {2 \cdot {\rm{CV}}feature{s_A}}} \cdot 100 & \quad \quad [\% ] \cr} $$

This bidirectional matching is important because the matching process (e.g., using Lowe’s ratio test and RANSAC) can produce slightly different results depending on the direction. Combining the results from both directions helps mitigate asymmetries in the matching process and provides a more balanced and reliable final score. For each database entry, a score is calculated using the same postmortem query set and stored in a list of scores. If multiple CT slices from the postmortem examination are matched with the antemortem CV database, each image receives its own list of scores.

Importantly, AKAZE doesn’t rely on artificial intelligence (AI) or machine learning. It uses mathematical functions to detect, describe, and match CV features, so it doesn’t require any training– in fact, training is not even possible. This makes it a fast, efficient, and reliable method for feature detection and matching in a wide range of images, with the distinct advantage that every calculation is mathematically traceable, unlike AI methods which often operate as “black boxes”. Furthermore, in the antemortem CV database, only the CV feature sets are stored, linked to a pseudonymized patient ID. No images are stored in the database. The CV features also do not allow for the reconstruction of the image. However, similar CV features can be recognized through the comparison of descriptors, which results in the identification of matching points.

### Evaluation

**Fig. 2:**
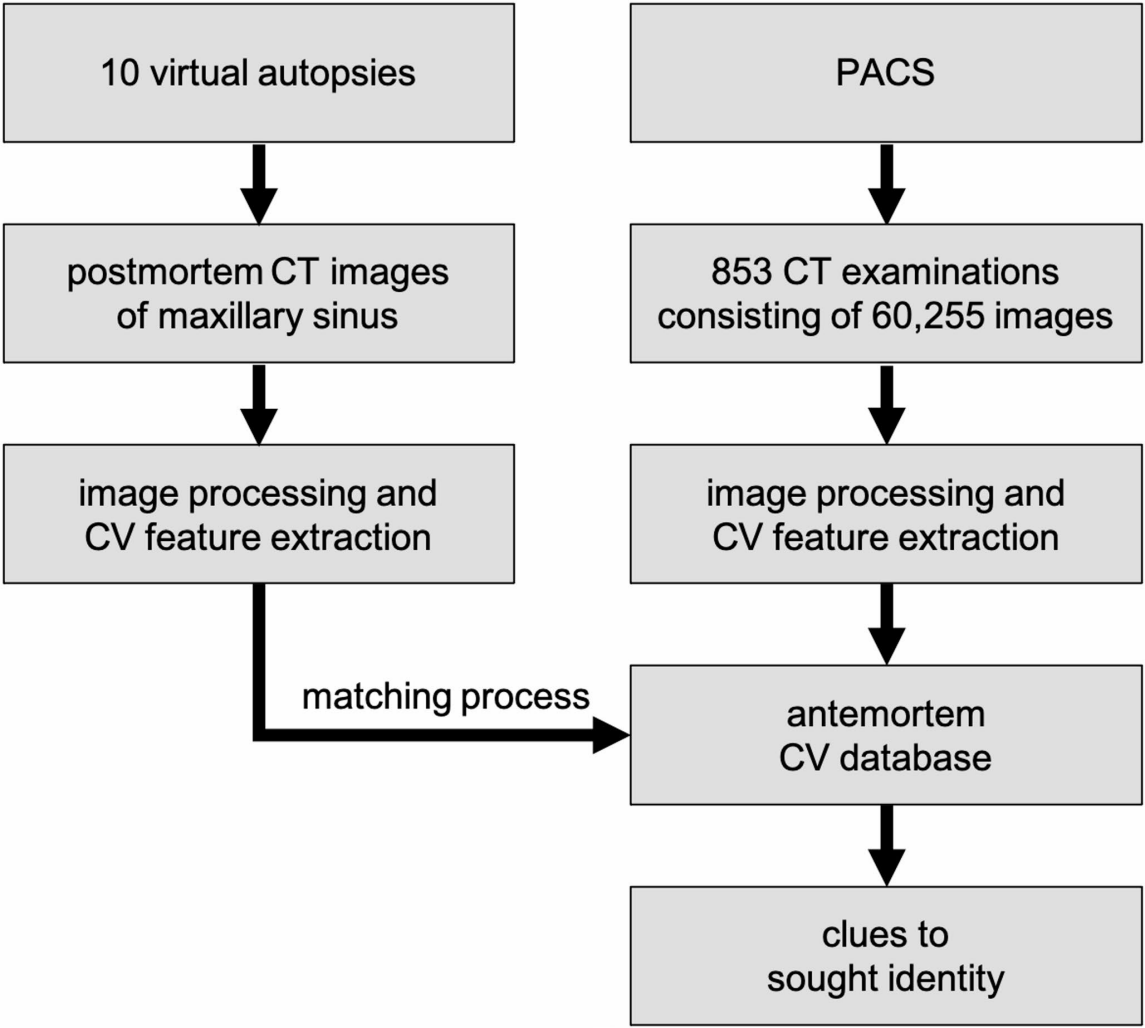
For 10 virtual autopsies, CT images of the maxillary sinus were selected for each case. CV features were extracted and compared with antemortem CV data to identify the 10 sought individuals. The antemortem CV database included CT slices from 853 examinations, exported from the picture archiving and communication system (PACS)

A total of 60,255 antemortem CT slices from 853 examinations of 738 individuals were processed and included in the CV database (see Fig. 2). For the postmortem images, the CT slice showing the largest representation of the maxillary sinuses was selected, along with slices covering the anatomical structures of the sinuses spanning 7.5 mm before and after it (equivalent to 12 slices at 0.625 mm thickness or 3 slices at 2.5 mm thickness). These 25, or in one case 7, postmortem slices from 10 identities were used to create query sets with CV features, which were then matched against the antemortem CV database. The parameters previously identified as optimal through systematic variation [10] were used: Sobel gradient 1.8, averaging filter 3, octaves 4, layers 4, diffusivity PM_G2, descriptor type KAZE, threshold 0.001, Lowe 0.6, and RANSAC 2.

### Identification process

For each comparison between a query set (representing a postmortem slice) and a single entry’s CV feature set from the antemortem database, a similarity score was calculated and stored in a list of scores. Each list, corresponding to a specific postmortem slice, contained 60,255 rows. In total, there were 25 or 7 lists per identification case, depending on the slice thickness. For each list, the highest score per identity was selected, resulting in 25 (or 7) filtered lists, each containing 738 scores corresponding to the 738 identities. A final consolidated list was created using one of the following two strategies:

- Score-based sorting: For each identity, the highest score was selected from all 25 (or 7) filtered lists. This list was then sorted in descending order based on the maximum scores. If scores were identical, the frequency with which an identity achieved rank 1 (where the score corresponds to the highest value in the filtered list) was considered first. If further differentiation was needed, the frequency of being in the top 10 across the filtered lists was taken into account.
- Rank-based sorting: Identities were sorted based on how frequently they achieved rank 1, followed by their frequency within the top 10 ranks across the filtered lists. Finally, if ties remained, their highest score was used for further sorting.

The consolidated list was then evaluated, with the position of the sought identity indicating at which rank the identification was successful (rank 1 corresponds to position 1, rank 10 corresponds to a top 10 position in the list).

## Results

In this study, for each identity from 10 virtual autopsies, 25 postmortem CT images (or in one case 7) showing the maxillary sinus were matched with an antemortem CV database containing 60,255 CV features sets from 853 examinations of 738 identities (see Table 2). The consolidated lists were then evaluated for each identification case, with the identification rate being 50% (5/10) at rank 1, 80% (8/10) at rank 2, and 100% (10/10) at rank 7 among the 738 potential identities for the rank-based sorting strategy (see Table 3).

**Table 2:** Demographics table of study participants from 853 CT examinations, which were added to the antemortem CV database

Age range [years]	Antemortem CV database			
	All	Female	Male	Unknown
10–19	15	3	12	0
20–29	53	10	41	2
30–39	73	9	64	0
40–49	78	19	54	5
50–59	107	34	71	2
60–69	108	33	66	9
70–79	151	80	65	6
80–89	206	111	82	13
90–99	60	19	32	9
100–109	2	0	2	0
all	853	318	489	46

**Table 3:** The table shows the position (ranging from 1 to 738) of the sought identity in the consolidated list, based on both the rank-based and score-based sorting strategies. It also indicates how often the sought identity achieved rank 1 across the query sets and provides the maximum score for all comparisons. For comparisons with other identities, the number of times a rank 1 position was occupied and the corresponding maximum score are included. In cases where multiple identities have the same maximum score, more than one identity May occupy rank 1. A comment is also provided to describe the challenges encountered during the identification process for both the antemortem (AM) and postmortem (PM) images of the sought identity

ID	Position of the sought identity in the consolidated list				Other identities		Comment
	Sorting strategy		Rank 1 [count]	Max score [%]	Rank 1 [count] (identities)	Max score [%]	
	Rank	Score					
1	1	1	15/25	2.25	4 (1x), 2 (2x), 1 (8x)	1.61	
2	2	3	4/25	1.38	5 (1x), 4 (2x), 3 (3x), 2 (2x), 1 (5x)	1.47	AM maxillary sinus injuries
3	1	10	8/25	1.36	3 (2x), 2 (4x), 1 (11x)	1.54	AM upper half of maxillary sinus visible
4	6	7	1/25	1.35	13 (1x), 5 (1x), 4 (3x), 1 (1x)	1.77	PM head displayed very small and AM upper half of maxillary sinus visible
5	2	1	12/25	1.73	13 (1x), 1 (4x)	1.61	
6	1	1	23/25	2.41	1 (2x)	1.67	
7	2	10	4/25	1.20	8 (1x), 3 (2x), 2 (5x), 1 (6x)	1.50	AM upper half of maxillary sinus visible
8	7	8	2/25	1.17	6 (1x), 5 (1x), 4 (1x), 3 (1x), 2 (3x), 1 (10x)	1.28	PM maxillary sinus injuries
9	1	1	25/25	1.94	1 (1x)	1.43	
10	1	1	4/7	2.85	1 (3x)	2.60	

**Fig. 3:**
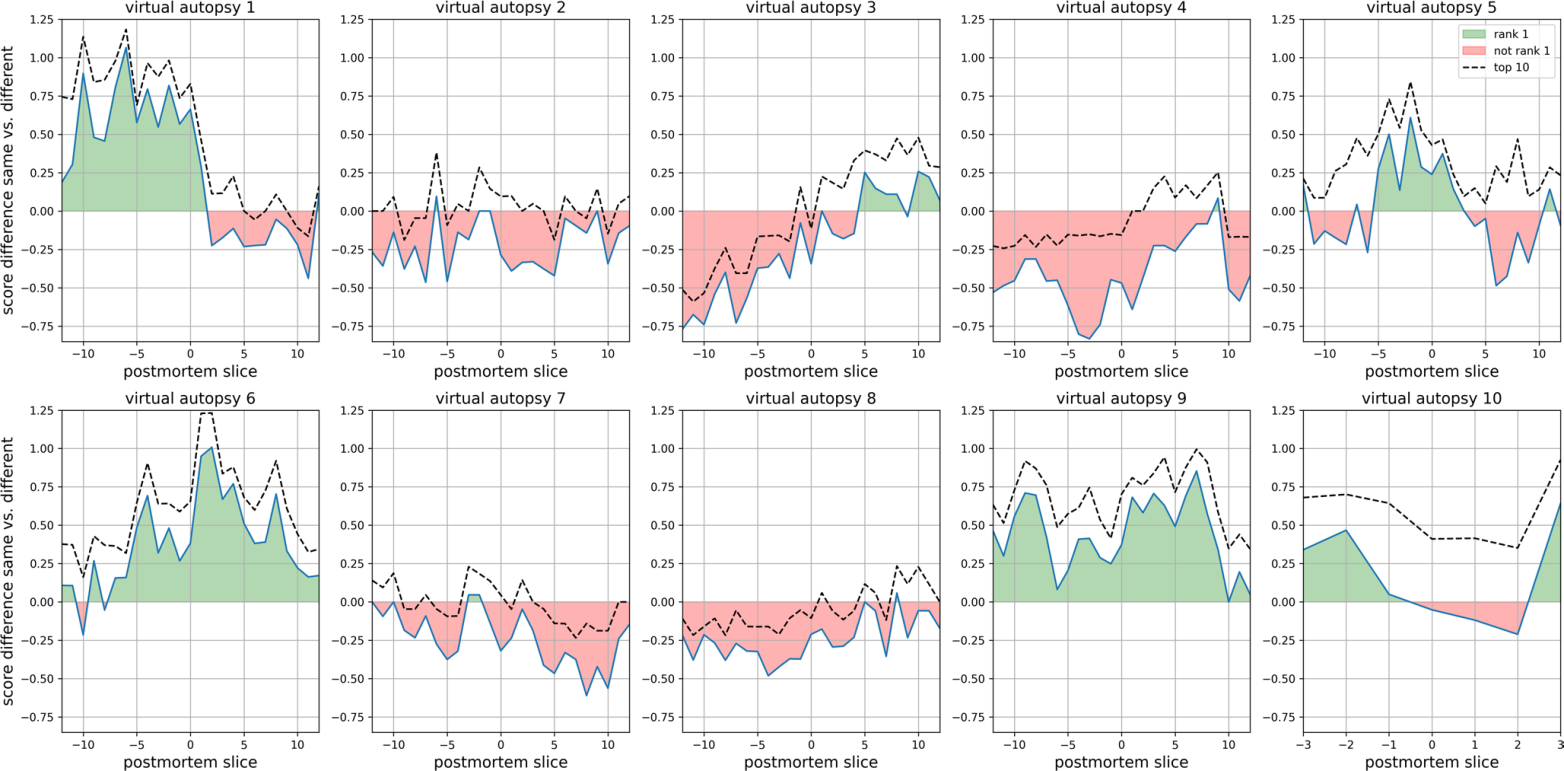
For all postmortem images compared with the antemortem CV database, starting from the CT slice showing the largest representation of the maxillary sinuses (x = 0), the maximum score difference between comparisons of the same (sought) identity and comparisons of different identities is calculated and presented. A positive value (green areas) indicates that the sought identity is ranked 1. Additionally, the maximum score difference at rank 10 is shown, indicating when the sought identity is among the top 10 identities, provided the value is greater than 0

In Fig. 3, the difference between the highest score of the sought identity and the highest score of a different identity is shown for all query sets (25 or 7 postmortem slices) and 10 virtual autopsies. Green indicates a clear rank 1 identification for the sought identity. It becomes evident that the selection of the postmortem slice influences the identification, as anatomical matches must be present between postmortem and antemortem slices. There were instances where the score for the sought identity was understandably lower, for example, due to maxillary sinus injuries or because only the upper half of the maxillary sinus was visible in the antemortem CT series.

**Fig. 4:**
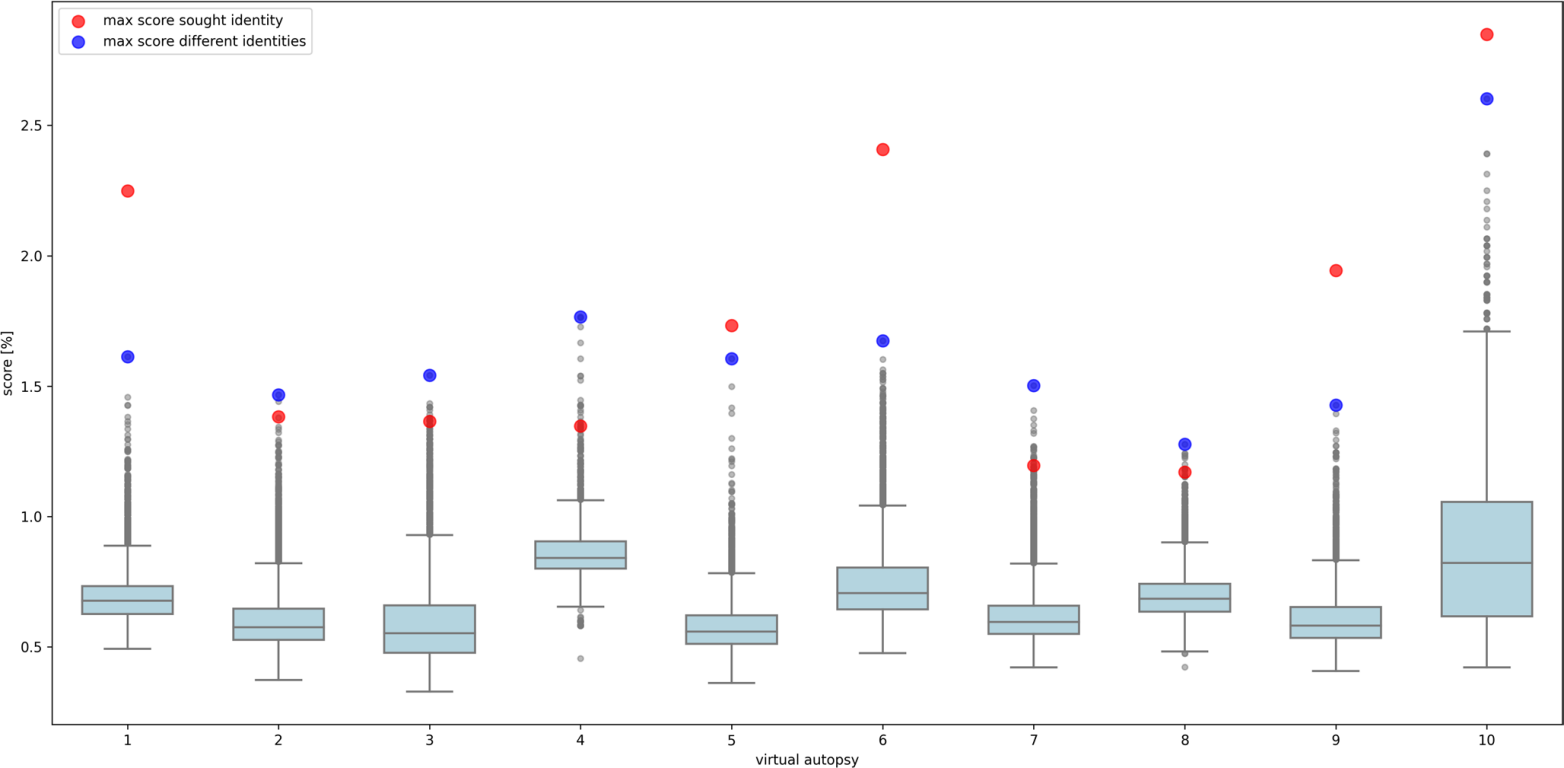
The image illustrates the results of CV-based personal identification across 10 virtual autopsies. Red and blue dots represent the maximum scores for the same individual and different individuals, respectively, across all results. The boxplots display the distribution of maximum scores for comparisons between postmortem and antemortem CV feature sets of different individuals. It is evident that the sought identity often achieves a higher score (indicating more matching points), facilitating identification. In this case, using the score-based sorting strategy, the sought identity matches the right individual in 50% of cases when only the highest score across all sets is taken into account

**Fig. 5:**
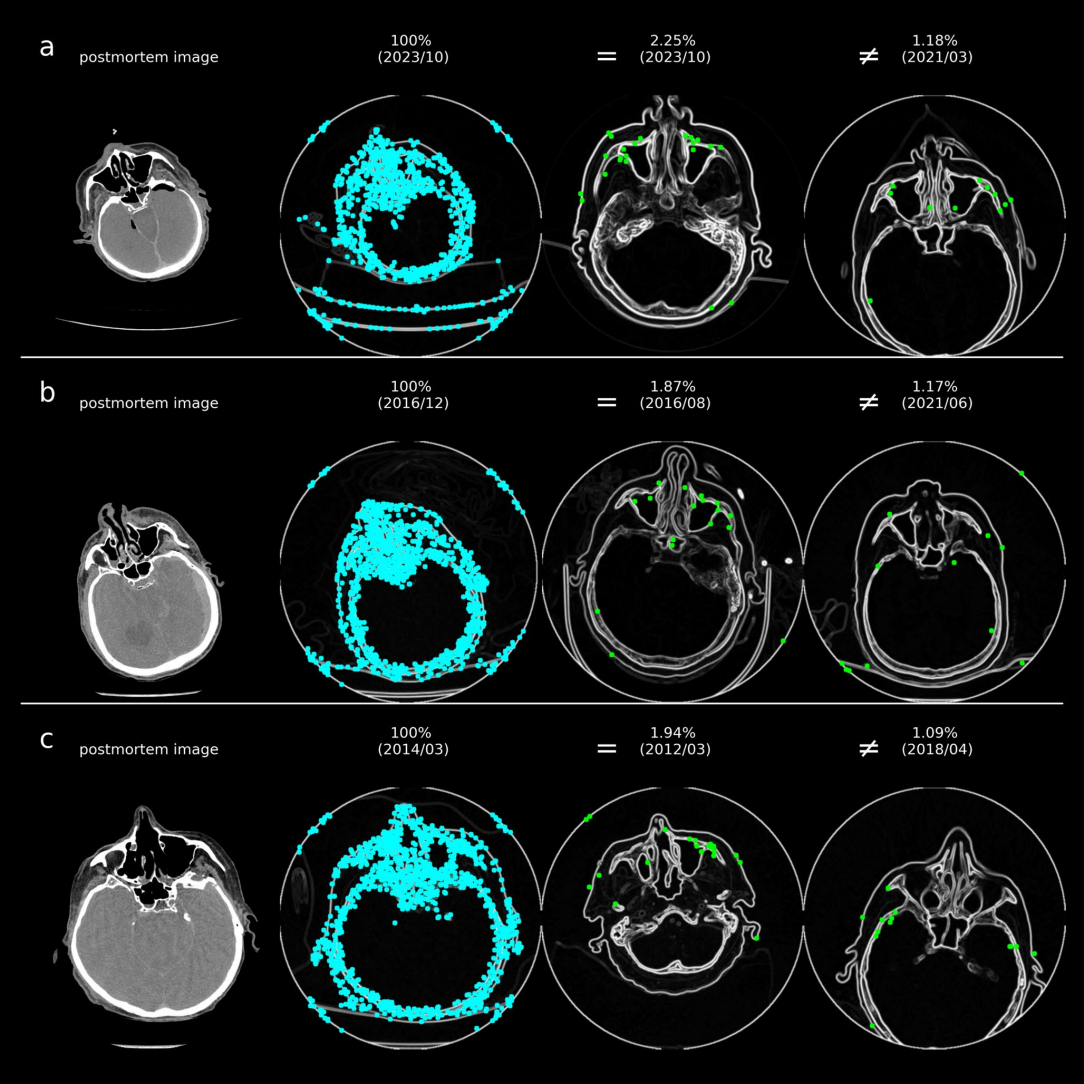
Examples of search images with CV features highlighted as blue dots (both left) and their best matches (green dots) for the reference image, either from the same identity (‘=’) or different identities (‘≠’, right), with scores displayed as percentages and the year/month of the image acquisition in parentheses. Matching points are often found within the maxillary sinuses, but they can also be identified in other areas, such as the skull bones. The results correspond to virtual autopsy cases 1 (a), 6 (b), and 9 (c) as shown in Figs. 3 and 4

In Fig. 4, the maximum scores for each identity across all comparisons are shown. The number of matching points was significantly higher for comparisons involving the same individual (1.76 ± 0.55%) compared to those involving different individuals (0.67 ± 0.15%), with 100% representing the maximum possible matching points. The sought identity generally exhibits a higher number of matching points compared to other identities, which facilitates a clear identification. However, matching points can also occur in comparisons between different identities, as certain anatomical structures may closely resemble each other (see Fig. 5).

**Fig. 6:**
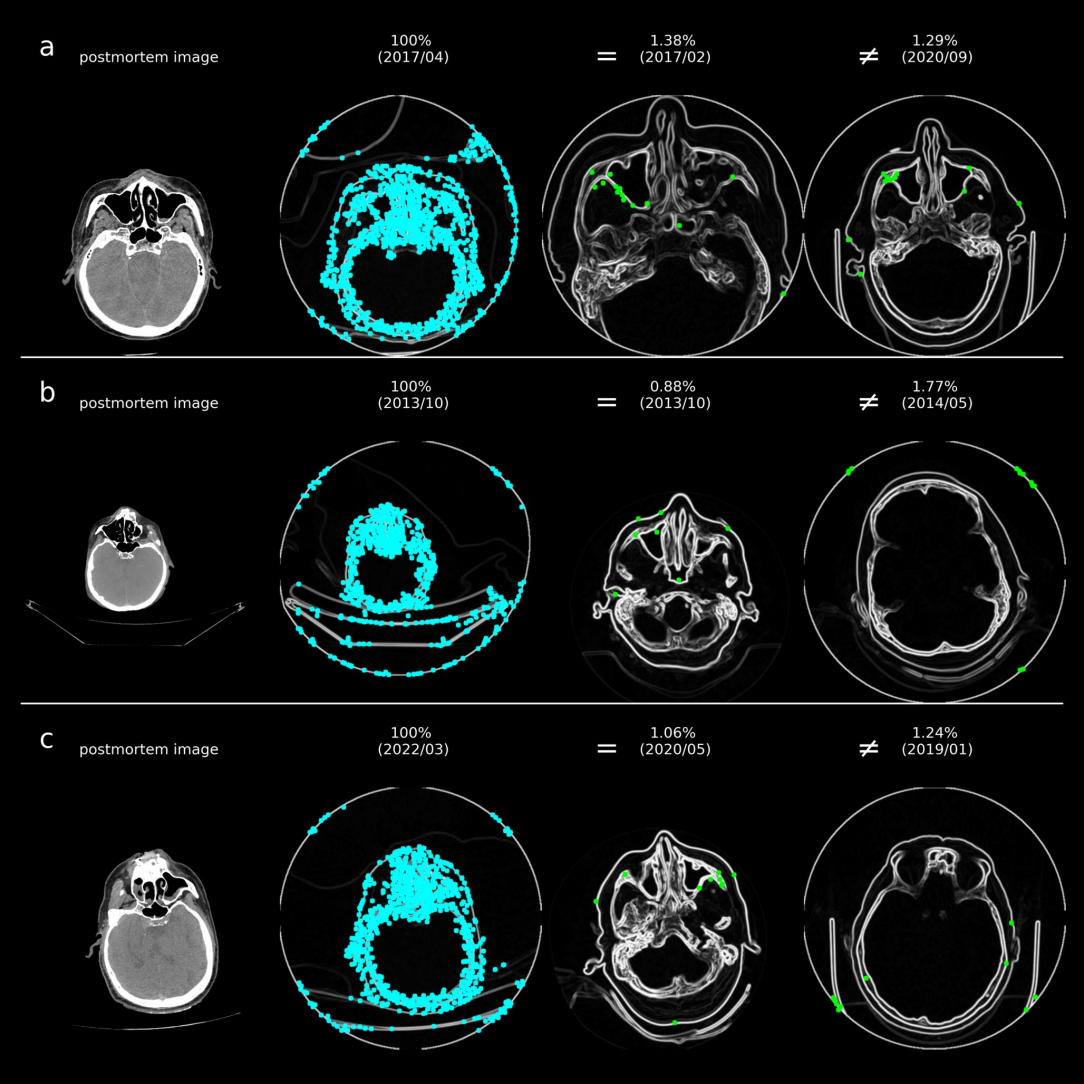
Examples of challenging identifications, with search images where CV features are highlighted as blue dots (both on the left) and their best matches (green dots) for the reference image, either from the same identity (‘=’) or different identities (‘≠’, on the right), with scores displayed as percentages and the year/month of the image acquisition in parentheses. The results correspond to virtual autopsy cases 2 (**a**), 4 (**b**), and 8 (**c**) as shown in Figs. 3 and 4. The identification process becomes more challenging when the maxillary sinus is not fully captured in the reference images, leading to other slices showing more matching points. Additionally, if the postmortem head appears smaller in the images due to an excessively large FOV, this can reduce the reliability of the matching points

In Table 4, the results for the individual antemortem reference examinations are presented. It is evident that, despite variations in CT parameters between the examinations, such as slice thickness, scan mode, and tube current, the sought identity could still be identified. The time span between the clinical (antemortem) and postmortem CT scans is indicated as the scan interval. Challenges arise when the antemortem reference CT examination only depicts parts of the maxillary sinus (due to the clinical question) or when injuries are present (see Fig. 6a and virtual autopsy 2 in Figs. 3 and 4). Another challenge occurs when the postmortem CT image of the maxillary sinus is extracted from a whole-body scan, where the head appears significantly smaller in the field of view (FOV) compared to clinical standards (see Fig. 6b and virtual autopsy 4 in Figs. 3 and 4). As a result, the focus may not always be on the maxillary sinus. In such cases, the skull bone may provide more matching points for the sought identity. An injury to the maxillary sinus in the postmortem image can also complicate automatic identification (see Fig. 6c and virtual autopsy 8 in Figs. 3 and 4). Additionally, for different identities, the boundary of the FOV in the CT image could present many matching points (compare with Fig. 6b and c on the right), leading to a higher score being measured than actually present.

**Table 4:** The results of the identification are presented, showing the position of the sought identity within the consolidated list, which is sorted by rank-based and score-based sorting strategies, separated for each individual reference series. For the sought identity, the number of rank 1 and positions within rank 10 are provided. Additionally, the slice thickness for the postmortem (PM) and antemortem (AM) slices is shown to demonstrate that the method still works with different CT parameters, as long as the displayed edges in the CT images are comparable. The scan interval refers to the time difference between the postmortem and antemortem CT examinations. A comment is also provided to describe the challenges encountered during the identification process

ID	Postmortem					Antemortem		Comment
	Position with sorting strategy		Rank 1 [count]	Rank 10 [count]	Slice thickn. [mm]	Slice thickn. [mm]	Scan interval [days]	
	Rank	Score						
1	257	254	0	0	0.625	2.5	2776	AM upper half of maxillary sinus visible
	2	1	6	7		2.5	6	
	1	1	15	22		5	6	
	23	4	0	5		5	6	AM part of maxillary sinus visible
2	2	3	4	18	0.625	2.5	47	AM maxillary sinus injuries
3	1	10	8	13	0.625	5	5	AM upper half of maxillary sinus visible
4	6	7	1	9	0.625	5	606	PM head displayed very small and AM upper half of maxillary sinus visible
	642	642	0	0		5	8	
	97	42	0	1		5	8	
5	2	1	12	25	0.625	2.5	96	
6	56	64	0	2	0.625	2.5	702	AM front half of the head visible
	1	1	18	25		5	375	
	1	1	8	13		2.5	276	
	1	1	12	21		2.5	276	
	1	1	18	25		2.5	275	
	1	1	18	25		3	227	
	1	1	20	23		2.5	137	
7	2	10	4	12	0.625	5	4	AM upper half of maxillary sinus visible
8	7	8	2	8	0.625	5	690	PM maxillary sinus injuries
	84	98	0	1		5	691	
9	1	1	25	25	0.625	0.625	739	
10	1	1	4/7	7/7	2.5	2.5	26	

## Discussion

This study demonstrates the feasibility of CV-based personal identification using postmortem CT images of the maxillary sinuses, achieving an identification rate of 50% at rank 1 (the sought identity is in the first position of the consolidated list), 80% at rank 2, and 100% at rank 7 (the sought identity is within the top 7 positions of the consolidated list) across 10 identification procedures and 738 potential identities. It confirms that the method from [10] is applicable not only to antemortem data, but also to postmortem data, which can be matched with complete CT series in an antemortem CV database. However, the score is lower when comparing postmortem and antemortem images of the sought individual, making identification more challenging.

Unlike the previous study [10], which exclusively analyzed antemortem CT images, the following differences can be observed: the score for comparisons of images from the same individual (sought identity) is significantly lower for postmortem-antemortem images than for antemortem-antemortem images (1.76% vs. 6.96%), whereas the score between different individuals remains comparable (0.67% vs. 0.64%). This discrepancy is not primarily due to osseous changes after death but rather to deviations from the clinical standard in head positioning, the relative size of the head within the FOV, and injuries, which result in fewer matching points being identified. In postmortem CT imaging, the body is typically placed on the CT table without fixing the head, unlike clinical CT scans, where the head is often positioned in a standardized manner. As a result, the alignment of the head in postmortem scans can differ significantly from the antemortem reference, leading to potential misalignments in the 2D slices. In fact, even small changes in head orientation can cause half of the image to show completely different structures. In previous study [10] difficulties in identification were already observed when head alignment varied, as only 2D slices are compared. Additionally, postmortem imaging may suffer from suboptimal conditions, such as an overly large FOV in relation to the head. This can result in much lower image resolution, further hindering the identification process. In addition, the antemortem references are not always optimal, as the maxillary sinus is not always fully included in the CT series, particularly in cranial emergency examinations. CV features rely on distinctive points, primarily edges. Injuries and variations in head orientation can alter these edges, leading to a loss of matching points. However, even small matching areas can still provide enough matching points for identification. The method was simplified by storing the full antemortem CT series in the antemortem CV database, while still reliably identifying the sought individual. Additionally, the virtual autopsies were retrospectively selected, with imaging conducted as part of routine forensic practice, not specifically for this identification method. Despite these challenges, identification using the CV-based personal identification method proved to be highly promising. There is still potential to further increase reliability by evaluating only database entries with similar characteristics, such as sex, approximate age [13], and estimated body weight, as the sought identity. Additionally, postmortem search images can be optimized by removing the boundary of the FOV, for example, by setting the gray value to air, which would eliminate many false matching points between different individuals. Alternatively, one could focus only on selected areas, such as the maxillary sinuses, and hide everything else.

Previous studies [7–9] have demonstrated the importance of paranasal sinuses, particularly the maxillary sinuses, in forensic identification. These structures are highly distinctive due to notable individual differences in their shape, size, symmetry, and contour, which align with the criteria for reliable identifiers: uniqueness, permanence, and immutability. Evidence supporting the use of paranasal sinuses for personal identification includes methods such as visual evaluations of CT examinations by multiple radiologists with different levels of experience [7], the comparison of morphometric data obtained from paranasal sinus measurements, and advanced analysis using techniques like iterative closest point algorithms applied to 3D reconstructions of the sphenoid sinus [8]. In contrast to traditional methods, this study demonstrates the feasibility of a completely different, fully automatable approach. Our results confirm previous findings, showing that, similar to human assessment, the CV algorithm is capable of identifying and recognizing numerous individual features. This research indicates that postmortem CT slices are sufficient for personal identification, and the entire process can be automated. In addition to the maxillary sinuses, the frontal sinuses have long been recognized as valuable for personal identification due to their unique morphology [14, 15]. Since 1926, they have been used in forensic casework [16]. A major challenge in conventional postmortem radiography has been achieving the correct alignment with the X-ray beam, often requiring multiple attempts to match antemortem images [17, 18]. Postmortem CT has significantly improved this process by enabling the reconstruction of virtual radiographs from CT data [18]. This approach allows for more precise comparisons with antemortem radiographs, reduces errors related to head positioning, and enhances accuracy in forensic identification. The CV-based personal identification method presented here is also suitable for comparisons between virtual radiographs and antemortem radiographs [19]. Additionally, differences in head orientation between postmortem and antemortem CT scans can be effectively addressed in the same way by selecting oblique slices from 3D CT data.

The CV-based personal identification is fundamentally not a legally secure method for identification [13]. Its task is rather to locate suitable reference materials and/or obtain clues to the sought individual. By narrowing down the possible identity from thousands of potential identities, a legally secure and forensic identification is subsequently facilitated with appropriate reference materials by a highly qualified professional. In this study, the professional would have had to review a maximum of seven different reference materials per case to identify the sought individual - out of 738 possible identities. This was achieved despite suboptimal postmortem imaging, injuries, and only partial representation of the maxillary sinus in the CT images. Under optimal conditions, identification was very clear (see Table 4). A visual inspection of the matching points in the images could have further reduced the number of possible identities beforehand (e.g., Fig. 6b-c, far right) or by filtering the data based on sex or age [13]. Studies using panoramic radiographs [20] and thoracic CT images [21] have shown that a threshold exists, indicating when an individual has been identified with high probability, eliminating the need for a full database comparison. This threshold is, for example, the maximum number of matching points derived from millions of image comparisons between different individuals. If the sought person exceeds this threshold, their identity is highly likely to be confirmed. For comparisons between postmortem images and clinical databases, similar thresholds are expected to exist. However, further evidence and methodological optimizations are needed to establish and refine these thresholds.

The AKAZE algorithm is a traditional CV method that employs predefined mathematical techniques to extract and match features based on image intensity and gradients, without relying on training or data-driven learning. This means the process is mathematically traceable. Furthermore, the CV features are abstract numerical features that cannot be used to reconstruct the original image. However, the greatest advantage is data privacy, which can address ethical and legal concerns. CV features can be decoupled from the image and, therefore, do not contain personal data. During a matching process, it can only be determined that the postmortem CV feature set corresponds with an antemortem CV feature set. Only a pseudonymized patient ID, linked to the antemortem CV feature set, allows a conclusion about the identity it pertains to. However, decryption, for example, can only be performed by the institution where the CT examination was conducted, thereby maintaining control of the data within the originating facility. Another approach would be that large CV databases do not contain patient IDs, but only the origin (clinic, practice) of the CV feature sets. In this case, no personal data would be present in the CV database. However, in the event of a high match, data from the origin could be further examined to identify the potentially sought individual.

The limitation of this retrospective study is that it primarily aims to demonstrate the potential of the CV-based personal identification method, without addressing ethical and legal considerations, which can be explored in future research. Additionally, the small sample size limits the ability to fully assess the method’s robustness, as only a limited number of postmortem cases were available in this study. Future studies could focus on collecting additional cases to build a larger population and address this limitation. For prospective studies, the main challenge is the need for very large CV database to ensure accurate identification rates. If the identity being searched for is not in the database, non-identification reflects the limitations of the data, not a flaw in the method itself. Additionally, the study was conducted at a single location, and ethnic differences may exist. The application of additional filters, such as age, sex, and estimated body weight, or the removal of interfering elements that cause incorrect matching points, such as limiting the FOV of the CT image, could further facilitate identification. Moreover, discrepancies in head orientation between postmortem and antemortem CT scans, which posed a challenge in this study, could be addressed in future research by selecting oblique slices from 3D CT data to minimize positional differences.

In conclusion, based on the findings, it appears possible to identify an individual using postmortem CT images from a virtual autopsy in conjunction with a clinical database. The CV-based personal identification method achieved 50% correct identification at rank 1, 80% at rank 2, and 100% at rank 7 across over 738 potential identities, even without the use of additional filters. However, for optimal results, postmortem imaging should ideally adhere to clinical standards to ensure sufficient CV matching points.

## Data Availability

The datasets analyzed during the current study are not publicly available due to privacy concerns related to the large volume of patient images. However, these datasets are available from the corresponding author on reasonable request, subject to appropriate ethical and legal considerations.
